# The Impact of the Hazard Correlation between Risk Factors and Diabetes

**DOI:** 10.3390/ijerph15102213

**Published:** 2018-10-10

**Authors:** Huan-Cheng Chang, Mei-Chin Wang, Meng-Hao Chen, Hung-Chang Liao, Ya-huei Wang

**Affiliations:** 1 Division of Nephrology, Department of Medicine, Taiwan Landseed Hospital, No. 77, Guangtai Road, Pingzhen Dist., Taoyuan 324, Taiwan; changhc@landseed.com.tw; 2 Department of Health Care Management, Chang Gung University, No. 259, Wenhua 1st Road, Guishan Dist., Taoyuan 33302, Taiwan; 3 Noble Health Management Center, Taiwan Landseed Hospital, No. 77, Guangtai Road, Pingzhen Dist., Taoyuan 324, Taiwan; wangmeria@landseed.com.tw; 4 Department of Medical Management, Chung Shan Medical University Hospital, No. 110, Section 1, Jian-Koa N. Road, Taichung 402, Taiwan; cshe845@csh.org.tw (M.-H.C.); yhuei@csmu.edu.tw (Y.-h.W.); 5 Department of Health Services Administration, Chung Shan Medical University, No. 110, Section 1, Jian-Koa N. Road, Taichung 402, Taiwan; 6 Department of Applied Foreign Languages, Chung Shan Medical University, No. 110, Section 1, Jian-Koa N. Road, Taichung 402, Taiwan

**Keywords:** diabetes, demographic variables, health exam variables, healthy behavior variables, environmental variables

## Abstract

This study examined the occurrence of diabetes and sustainable risk factors in residents aged 30 and above of a community in Taoyuan County, Taiwan. The main purpose of this research was to explore the correlations between related variables and the occurrence of diabetes. The demographic variables, health exam variables, healthy behavior variables, and environmental variables had obvious impacts on the risk of diabetes. As age increased, the risk of developing the disease also increased; higher educational levels lowered risk, while unemployment raised it. Also, analysis of the health exam variables showed that abnormal BMIs, waist-hip ratios, and body fat percentages had significant impacts on individuals’ risk of diabetes. Moreover, it was found that smoking affected the risk of having diabetes: smokers, particularly male smokers, had a relatively higher risk of developing the disease. Lastly, the results showed that exposure to second-hand smoke did not have a significant effect on the diabetes proportion in the male population. However, a significantly higher proportion of females who had been exposed to second-hand smoke had diabetes.

## 1. Introduction

Due to changes in lifestyle, the worldwide occurrence of non-communicable diseases is increasing rapidly, and with it, the urgency to confront and focus on the prevention of such diseases. Consequently, many recent studies focus on such diseases, including diabetes, and their contributing factors. Diabetes—a disease attributable to a disorder of the metabolism—is the result of insufficient hormones (commonly called insulin) secreted by pancreatic β cells or nonfunctioning β cells. The secretion of insulin helps digested glucose enter the body’s cells to be used as energy. If the secretion is insufficient, or if the β cells are nonfunctioning, the glucose will remain in the blood stream or be discharged in urine [1]. Early symptoms of diabetes, which include feeling thirsty, urinating often, feeling hungry, and losing weight, are not easy to detect [2]. Having the disease for a long time without treatment may lead to physical injury, dysfunction, or even organ failure, especially organs such as the eyes, kidney, nerves, heart, and blood vessels [3]. Complications such as retinopathy, renal disease, neuropathy, and vascular disease, if not managed properly, will lead to heart disease, strokes, or other complications related to the eyes, kidneys, and vascularity of the lower extremities, requiring difficult treatments such as dialysis, extreme interventions like amputations, and in some cases, leading to irreversible disabilities such as blindness or even death [4].

A 2012 World Health Organization (WHO) report revealed that of the estimated 57 million global deaths in 2008, 36 million (63%) were due to non-communicable diseases—specifically, in order of prevalence, cardiovascular disease, cancer, chronic respiratory diseases, and diabetes. The report also indicated that one third of the world’s adults suffer from high blood pressure, while one tenth suffer from diabetes [5]. The International Diabetes Federation (IDF) also estimated that the global diabetes population in 2012 exceeded 371 million, with nearly half of this number unaware that they were suffering from the disease. There were 4.8 million deaths from diabetes in 2012, while the annual total of global diabetes-related medical costs that year was estimated to be 471 billion USD. The top ten nations with the most diabetes patients in 2012 were China, India, the U.S., Brazil, Russia, Mexico, Indonesia, Egypt, Japan, and Pakistan, with 92.3, 63, 24.1, 13.4, 12.7, 10.6, 7.6, 7.5, 7.1, and 6.6 million patients, respectively. The number of diabetes diagnoses is increasing annually all over the world [6].

Due to demographic, lifestyle, and diet changes in recent years, diabetes has become the fourth leading cause of death in Taiwan, with the patient population now in excess of 1 million [7]. Statistical data from 2011 showed that the total diabetic population in Taiwan that year was 1,589,097 (803,888 males and 785,209 females), accounting for 6.85% of the total population. The data from the same year revealed that, in terms of clinic visits, males outnumbered females in the 0–14 and 30–64 age groups, while the female population was higher in the other age groups. The 75–79 age group had the highest rate of clinic visits for both genders. Studies have shown that the rate of diabetes for citizens over the age of 19 was 5% in 1997, but by 2002, it had risen to 8% [8]. A 2006 report from Taiwan’s Ministry of Health and Welfare demonstrated that unless healthy diet and exercise habits are undertaken and weight is controlled, the likelihood of patients with early symptoms having full-blown diabetes within a year is 16.4% for males and 13.6% for females, which means that one out of every seven people will have diabetes [9].

Over the past few decades, empirical research has been devoted to the investigation of the relationship between diabetes and other factors. In 2007, to study the risk of diabetes, Dankner et al. [10] conducted a progressive research study on diabetes to predict the 20-year diabetes rate in Israel. From 2003 through 2004, Dankner et al. administered diabetic testing of 935 people, none of whom had diabetes in 1980. After deducting the 263 people who had passed away, the results showed that 25.9% (174 people) of the remaining 672 people tested had become diabetic patients, of whom 104 (59.8%) were male and 70 (40.2%) were female. Similarly, Harati et al. [11] published a six-year cohort study on diabetes in Iran. The results showed that 237 people tested who did not have diabetes became diabetic during the six-year period. The six-year cumulative incidence rate (CIR) was 6.4% (confidence interval (CI): 5.6–7.2), with an annual incidence rate of 10.6% (CI: 8.2–12.1).

González et al. [12] conducted a diabetes survey in the U.K. from 1996 to 2005. The results showed that the rate of diabetes prevalence in the U.K. rose steadily, from 2.71% (CI: 2.58–2.85) in 1996 to 4.42% in 2005; diabetes prevalence rates in the male population were higher than those in the female population in any age group. According to Boyle et al. [13], the diabetes incidence rate in the U.S. was 3.3% in 1980, and it climbed to 7.8% by 2007. Lysy et al. [14] conducted a study in 2012 on diabetes data collected from April 2006 to March 2007 in Ontario, Canada. The results showed that 88,886 new diabetes cases occurred during that year, of which 47,091 were male (51.98%) and 41,795 were female (47.02%); the average age was 59.14, and the prevalence rate was 8.26% (CI: 8.20–8.31).

In 2004, Wild et al. [15] conducted a Year-2000 diabetes prevalence rate study on the 191 member states of the WHO, and forecasted a 2030 diabetes prevalence rate based on the results of that study. After standardizing the age variable, the diabetes prevalence rate in 2000 was 2.2%, with the forecasted rate expected to rise to 4.4% by 2030; the prevalence rate for men was higher than that for women. However, the number of women with diabetes was higher than the number of men. In 2009, Shaw et al. [16] estimated the diabetes prevalence rate for the period of 2010 through 2030 for 216 countries. The results estimated the global prevalence rate variation for the 20–79 age group to be 6.4% for 2010, rising to 7.7% by 2030.

Zabetian et al. [17] conducted a literature review in 2013, collecting the literature data on diabetes in the Middle East for the period from January 1990 through January 2012. The findings showed that the crude prevalence rates in Middle Eastern countries for the 30-year period ranged widely from 2.5% in 1982 to 31.6% in 2011. The IDF [18] updates its global data on diabetes annually. According to the 2012 report concerning the relative prevalence rate in the 20–27 age group, the top 10 countries in the world were: Kiribati (25.7%), the Republic of the Marshall Islands (22.2%), Kuwait (21.1%), the Republic of Nauru (20.7%), Lebanon (20.2%), Qatar (20.2%), Saudi Arabia (20.0%), Bahrain (19.9%), Tuvalu (19.5%), and the United Arab Emirates (19.2%), most of which are located in the Middle East and North Africa (MENA) region or the Pacific region. Further analysis of the 2012 global diabetes data updated by the IDF [19] in 2013 revealed that seven of the top 20 countries in the world with the highest diabetes prevalence were located in the MENA region. In addition, seven countries were located in the Pacific region, four countries in the North American region, and four countries in the Caribbean region. The data also showed that the relative prevalence rate in Taiwan was 8.55%, ranking it 87th globally. However, this rate was still higher than the global relative prevalence rate of 8.2%. In addition, diabetes now ranks fourth in the top ten causes of death in Taiwan, with the diabetes patient population reaching one million [20].

One major danger of diabetes is that the symptoms usually occur slowly, making it difficult to detect. While the causes of diabetes are related to personal habits and family history, living environment is also an important factor. Exposure to cigarette smoke, for example, increases the risk of diabetes. Many studies indicate that smoking is an independent risk factor for diabetes development [21,22]. Zeilinger et al. [23] first reported that smoking plays a significant role in the gene methylation pattern. According to published international diabetic information, the risk factors that are commonly attributed to the cause of diabetes are family history, age, obesity, unhealthy diet, lack of exercise, and high blood pressure. The WHO [24] also lists smoking as a risk factor for diabetes, based on the research findings that the nicotine in cigarettes is the main component that heightens a smoker’s risk of diabetes.

Most of the previous studies on diabetes described the impact of sustainable risk factors and diabetes. The following studies have had similar results with regard to passive smoking. Pan et al. [25] estimated that 11.7% of diabetes cases among men and 2.4% of diabetes cases among women would be caused by active and passive smoking. Furthermore, Ko et al. [26] studied the impact of exposure to a passive smoke environment on the diabetes prevalence rate, specifically among people who had never smoked. The findings showed that exposure to passive smoke was an important risk factor for diabetes. Hayashino et al. [27] also carried out a cohort study on passive smoke exposure and diabetes occurrence among manual laborers. The results also suggested that exposure to passive smoke was one of the risk factors for having diabetes. The risk ratios revealed that the difference in the risk of having diabetes between smokers and nonsmokers who had been exposed to a passive smoke environment was not significant. Zhang et al. [28] conducted research on passive smoke exposure and the prevalence of diabetes among women, finding that long-term exposure to a passive smoke environment significantly heightened the risk of diabetes.

Characteristics of the living environment are also important factors. The identification of community characteristics and personal habits is essential in improving health care and public policy in order to reduce the prevalence and age of onset of the disease among the wider public [29]. Therefore, this study used the diabetes database maintained by a community hospital in Taoyuan County in Taiwan as the source of demographic and health data. The study focused on the results of a community health assessment administered in 18 neighborhoods in the region to determine the status of diabetes in the area and explore the sustainable risk factors related to diabetes. This study adopts this topic as the research theme, exploring the relationship between risk factors and diabetes. The study results are offered as a reference for formulating policy to improve community health programs and prevent disease.

Given the theoretical positions taken for the study and the status of the field, the study aims to answer the following questions:

1. Is there a significant relationship between demographic variables and the occurrence of diabetes?
2. Is there a significant relationship between health exam variables and the occurrence of diabetes?
3. Is there a significant relationship between healthy behavior variables and the occurrence of diabetes?
4. Is there a significant relationship between environmental variables and the occurrence of diabetes?

## 2. Resources and Methodology

### 2.1. Resources

This study examined diabetes statuses and sustainable risk factors among a sample population of residents aged 30 and older of a community in Taoyuan County, Taiwan. The research data were drawn from the diabetes database maintained by a community hospital in the county. A total of 6057 people were included in this study by the end of November 2011. The number of valid samples included in the study after screening was 5886. The main purpose of this research was to explore correlations between certain lifestyle and physiological factors, and the occurrence of diabetes. The lifestyle and physiological variables examined were demographic details, responses to health exam questions, and healthy behaviors. The collected demographic details included gender, age, levels of education, employment, and family income. The health exam items included BMI, Waist-Hip Ratio, Body Fat Percentage, and Hypertension. The healthy behaviors were obtained from the yes-no questions of the samples’ self-perceived habits, including exercise habits, smoking habits, and drinking habits. Environment situations were explored by whether the samples were exposed in the hazard of second-hand smoking.

### 2.2. Analysis Methods

This study used the statistical software SPSS22.0 (IBM Corporation, Armonk, NY, USA) for Windows to perform the research analyses. The chi-square analyses were performed to identify whether correlations existed between any of the independent variables (demographic details, health exam answers, healthy behaviors, and environment situations) and the dependent variable (having diabetes). A logistic regression analysis was performed to examine whether second-hand smoke had a significant impact on the occurrence of diabetes. The proportional testing was used to explore the differences in percentages of the populations with diabetes in each variable group.

## 3. Results

### 3.1. The Correlation Analysis between Each Demographic Variable and Diabetic Patients

This study explored the correlation between each demographic variable and diabetic patients, using the diabetes database maintained by a community hospital in Taoyuan County of Taiwan. Table 1 shows that every variable in the demographic variable group was significantly correlated with having diabetes. First, the results showed that gender was significantly correlated with having diabetes (χ^2^ (1) = 4.103, *p* = 0.043). Further, checking using proportional testing (see Table 2) confirmed that the likelihood of having diabetes differed between the genders, and that males were more likely to suffer from the disease than females: 12.39% of the male population had been diagnosed with diabetes, while only 10.70% of females had been.

Second, the results showed that age was significantly correlated with having diabetes (χ^2^ (4) = 215.08, *p* < 0.001). The overall trend showed that as age increased, the likelihood of having diabetes also increased. The percentages of diabetes diagnoses for each age group were as follows: for the 30–44 age group, 3.16% of the population had diabetes; for the 45–54 age group, 7.62%; 55–64, 14.23%; 65–74, 19.74%; and for those aged 75+, 20.6%.

Third, the results showed that an individual’s level of education was also significantly correlated with having diabetes (χ^2^ (4) = 131.434, *p* < 0.001). However, the trend of having diabetes in this variable group ran contrary to that of the age variable—the higher the level of education attained, the less likely that individual was to have diabetes. The percentage of the “illiterate” subgroup with diabetes was 22.76%, while only 6.62% of the “college-educated and above” subgroup had the disease.

Fourth, the results showed that employment was significantly correlated with having diabetes (χ^2^ (1) = 125.043, *p* < 0.001). Further examination using proportional testing (see Table 2) revealed that the percentage of people with diabetes was higher in the unemployed subgroup (16.62%) than in the employed subgroup (only 6.67%). 

Lastly, in terms of family income, the chi-square testing results showed that family income was significantly correlated with having diabetes (χ^2^ (3) = 29.484, *p* < 0.001). Among the subgroup that earned less than NT$800,000 per year, the percentage of people with diabetes was 13.4%; in the “800,000–1 million” subgroup, 8.8% of the population had diabetes; in the “1–1.3 million” subgroup, the percentage was 7.4%; and in the “1.7+ million” subgroup, the percentage was 8.1%. When graphed, the population spread formed a slight U-shape.

### 3.2. The Correlation Analyses between Health Exam Variables and Having Diabetes

Using Chi-square testing, the statistical results showed that all of the health exam variables were significantly correlated with having diabetes (see Table 3). In terms of diabetes prevalence, people with “higher than normal” exam results were more likely to have diabetes. BMI readings were found to be significantly correlated with having diabetes (χ^2^ (1) = 90.497, *p* < 0.001). The diabetes population in the group whose BMI readings exceeded the normal range was 16.31%, while among those with normal BMI readings, it was 8.24%.

The results showed that an individual’s waist-hip ratio was also significantly correlated with having diabetes (χ^2^ (1) = 208.939, *p* < 0.001). Among the population whose waist-to-hip ratios fell within the normal range (male <0.9; female <0.8), the percentage of diabetes patients was 7.86%, whereas among those whose waist-to-hip ratios were higher than normal, the percentage of diabetes patients was 21.46%.

In terms of body fat, the results showed that a high body fat percentage was also significantly correlated with having diabetes (χ^2^ (1) = 28.307, *p* < 0.001). The percentage of the population with higher-than-normal body fat who had diabetes was 13.64%, while 9.24% of people with normal body fat had diabetes.

The results showed that hypertension was significantly correlated with having diabetes (χ^2^ (1) = 64.286, *p* < 0.001). Of the population with hypertension, 21.08% suffered from diabetes; this percentage was reduced by half among those without hypertension, with 10.31% of this subgroup having the disease. Proportional testing (see Table 2) was also done on each health exam variable, and the results showed that the percentages of diabetes patients in each subgroup of each variable differed significantly, and that individuals with hypertension had a higher likelihood of having diabetes.

### 3.3. The Correlation Analyses between Healthy Behavior Variables and Having Diabetes

We looked at the results of the chi-square analyses of the healthy behavior variables and found that of the three lifestyle variables that we examined, only habitual smoking was significantly correlated with having diabetes (see Table 4). The results showed that exercise habits were not significantly correlated with having diabetes (χ^2^ (1) = 0.000, *p* = 0.999). The result for the smoking variable was (χ^2^ (2) = 8.138, *p* = 0.004), however, which showed that habitual smoking was significantly correlated with having diabetes. Of the subgroup of smokers, 16.89% suffered from diabetes, while only 11.95% of non-smokers did. Lastly, the test result for the drinking habits subgroups was (χ^2^ (2) = 0.091, *p* = 0.762), which showed that drinking habits were not significantly correlated with having diabetes.

### 3.4. The Correlation Analyses between Environmental Variable and Having Diabetes

According to the statistics, the research samples of this study totaled 5886, with 2680 men and 3206 women; male diabetic patients totaled 332, and female diabetic patients totaled 343. Table 5 shows that 14.17% of the diabetic population had been exposed to second-hand smoking environments, while 11.95% the diabetic population had not been exposed to second-hand smoking environments. The proportion test showed that the two groups had a significant difference. In terms of gender, after performing the proportion test, the results showed that having been exposed to a second-hand smoking environment did not have a significant effect on the diabetes proportion in the male population. For the female population, there was a significantly higher diabetes proportion among those who had been exposed to second-hand smoking environments (13.65%).

In addition, this study performed a logistic regression analysis. Table 6 shows the analysis results. The analysis of the overall population showed that the risk of having diabetes for those who had been exposed to second-hand smoking environments was higher than for those who had not been exposed to second-hand smoking environments. The odds of having diabetes for those who had been exposed to second-hand smoking environments were 1.389 times higher than for those who had not been exposed (95% Confidence Interval (CI): 1.172–1.647). In terms of gender, the results showed that those who had been exposed to second-hand smoking environments had higher risks of having diabetes than those who had not been exposed. In the male population, the odds of having diabetes for those who had been exposed to second-hand smoking environments were 1.301 times higher than for those who had not been exposed (95% CI: 1.034–1.636), while the odds for the female population were 1.532 times higher (95% CI: 1.144–2.053).

## 4. Discussion

Chi-square testing revealed that each demographic variable was significantly correlated to being diabetic. The first demographic variable examined was gender. Berg-Kelly discussed the issue of gender and disease in 1999, proposing that the factors are indeed correlated, but that the correlation may be affected by other factors, such as physical characteristics, social climate, environment, and individual perceptions [30]. Doyal also mentioned in 2001 that in some respects, men and women face different risks when it comes to disease, and that, in addition to the effects of different physical characteristics, the differences in gender awareness imposed by the community also contribute to the results [31].

Therefore, it is necessary to control for gender in epidemiological studies. In this paper, the results of the chi-square testing indicated that being male was significantly correlated with having diabetes, as the percentage of males with diabetes was 12.39%, noticeably higher than that of females (10.70%). Although this result is similar to most recent findings, we cannot conclude that the significant correlation between gender and having diabetes is absolute. Rather than claiming that simply being male increases an individual’s risk of developing diabetes, there is evidence to support the idea that the different living habits of each gender contribute to the different chances of having diabetes. Choi et al. researched race and gender differences in diabetic patients over 18 years old in Californian Asian communities in 2009 and found that even with living habits and other related risk factors under control, gender and race were still correlated with having diabetes. After testing, the research results showed that significant correlations only existed among the white and Hispanic populations; however, the percentage of males who had the disease was still higher than that of females [1]. Nonetheless, in other countries, females are also likely to get diabetes. Silverston et al. [32] surveyed the population of Anniston community (Alabama, USA) to investigate the correlation between polychlorinated biphenyl exposure and diabetes. They found that there is a significant correlation between elevated levels of polychlorinated biphenyl and diabetes, especially in the situation when the subjects are female and those less than 55-years old. Manjoo et al. [33] examined current obesity thresholds signifying diabetes risk. The participants were an aboriginal population in the *Cree of Eeyou istchee* of northern Quebec, Canada. The study concluded that those exposed to the following two situations will exhibit an increased diabetes risk: One is any male or female that has a BMI of 30 kg/m^2^ or above; the other is that a male has a waist circumference of 102 cm.

Interestingly, the research conducted by Jiang et al. in 2012 on diabetes prevalence and occurrence rates using the 2000–2009 National Health Insurance data yielded similar findings. Their results showed that, concerning the annual prevalence rates in males and females, the female prevalence rate was consistently higher than that of males before 2008, at which point the trend reversed, becoming higher in males [34]. Although the cause of this gender difference is unknown, the results of this study were consistent with those of various previous studies in finding that the correlation between gender and having diabetes is strong but not absolute. The strength of the correlation may be affected by time, the attitudes common among the population, or by changes in lifestyle.

In terms of age, the IDF has identified aging as a risk factor for diabetes [6], and Taiwan’s Ministry of Health and Welfare has further identified that individuals over 45 years of age are considered at high risk for developing diabetes [9]. In the results of this study, age was also significantly correlated with having diabetes: as age increases, the likelihood of having diabetes increases. Looking at the male group alone, the subgroup with the highest percentage of diabetes diagnoses was the 65–74 age group; however, the percentage of the 75+ age group was still higher than all age groups younger than 64. Therefore, the results can still be considered to match the diabetes risk factors identified around the world.

Levels of education were significantly correlated with having diabetes. Among the male population, the subgroup with the highest percentage of diabetes diagnoses was the “literate or elementary school” group rather than the “illiterate” group; however, the percentage in the “illiterate” group was still higher than that of every group above “high school.” The Taiwan National Health Survey of 2005 also produced results similar to this study: the lower the educational level, the higher the prevalence rate (18.3% in the illiterate group, 11.4% in the literate or elementary school group, 4.8% in the junior high school group, 2.5% in the high school group, and 1.5% in the college and above group) [35].

Concerning the correlation between educational levels and diabetes, Kenkel has explored the relationship among healthy behaviors, health knowledge, and school education. Kenkel’s study demonstrated that the positive correlation between education and health has already been proven, and that education can be used to increase health-related knowledge among the public, thus improving living habits, promoting a healthy quality of life, and lowering the risk of developing diseases [36]. Buckley et al. conducted research in 2004 concerning the impact of socioeconomic factors on the health of the senior population in Canada; their findings also revealed a positive correlation between educational levels and health [37].

Additionally, being unemployed was significantly correlated with having diabetes. When the percentages of the two subgroups with diabetes were compared, the number of diabetic patients was clearly higher in the unemployed subgroup than in the employment group. There may be a number of reasons for this difference. Unemployed people may display relatively less daily activity and are more likely to have relatively depressed moods, both of which can increase the risk of developing diabetes. Related studies conducted domestically and abroad concerning this issue are very sparse, but a few have been published. In their study on unemployment and health, Reine el al. explained that employment has a correlation with health, and that the impact was particularly great on younger people [38]. Friis et al. also explored the association among unemployment, depression, and diabetes. Their findings showed that there was a correlation between employment and diabetes, and that unemployed people had a higher probability of having diabetes [39]. A related factor is family income, which is also significantly correlated with having diabetes. The likelihood of having diabetes decreased as family income increased; however, when family income reached a certain level, the percentage of the diabetic population rose again. The trend formed a U-shape when depicted on a graph. The 2005 Taiwan National Health Survey discovered a similar phenomenon: as income levels decreased, the diabetes prevalence rate became higher, but the prevalence rate was also high at the highest income level (when monthly income was less than 10,000, the rate was 7.6%; less than 20,000, 5.1%; less than 40,000, 2.6%; less than 60,000, 2.4%; and more than 60,000, 4.6%) [35]. Buckley et al.’s 2004 research found that income levels and chronic illness were correlated, and that people with higher incomes were generally in better health. Buckley et al. speculated that people with higher incomes could afford regular medical care, and that they are generally better educated [37]. Humphries et al. conducted research on the relationship between income and health. Their findings showed that people with higher incomes had a higher perceived health status, while those with lower incomes had a lower perceived health status. That is, the higher an individual’s income level, the better his or her self-perceived health status was [40].

All of the variables covered on the health exam were significantly correlated with having diabetes. BMI, waist-hip ratio, and body fat index are all obesity indicators. They were divided into higher-than-normal and normal for analysis purposes in this study, in which higher-than-normal means overweight or obese. The results showed that BMI and waist-hip ratio were significantly correlated with having diabetes, and people with higher-than-normal readings were more likely to have diabetes. The IDF identified obesity as a risk factor for having diabetes [6]. The WHO takes the same view and indicates on its website that studies show that an elevated BMI is a major indicator and risk factor for developing non-communicable diseases such as cardiovascular disease, diabetes, musculoskeletal disorders, and certain cancers [5]. In addition, Taiwan’s Ministry of Health and Welfare further identified obese individuals as a “group at high risk” or “the group at highest risk for diabetes” [9]. It is worth mentioning that waist-hip ratio is a better indicator than BMI in predicting diabetes. Cheng, et al. conducted research to determine whether waist-hip ratio was a better indicator for the disease: their results showed that even though BMI is an effective indicator in predicting diabetes, waist-hip ratio is more effective in predicting the risk of developing diabetes [41]. The results of this study confirmed their findings.

The IDF also identified hypertension as one of the risk factors for diabetes [6], and the ADA (American Diabetes Association) mentioned in its 2011 report, “Diagnosis and Classification of Diabetes Mellitus,” that people with hypertension should undergo regular screening for diabetes for this reason [42]. The Taiwan’s Ministry of Health and Welfare also identified people with hypertension as being at high risk of diabetes [9]. This study echoed these findings, proposing that hypertension is significantly correlated with having diabetes. When the sample population was divided into two subgroups according to whether or not they had hypertension, the percentage of diabetic patients was much higher in the hypertensive subgroup than it was in the non-hypertensive subgroup, thus confirming the result of the IDF, the ADA, and Taiwan’s Ministry of Health and Welfare, that hypertension is a risk factor for diabetes.

In light of the health exam variables, the results indicated that only smoking was significantly correlated with having diabetes. Conventional thinking and relevant research both assert that exercise is beneficial to physical health, and can effectively reduce the occurrence of diseases [43]. Thus, Taiwan’s Ministry of Health and Welfare classifies people who do not exercise regularly as comprising a population at high risk of developing diabetes [9], as do the WHO and other organizations [5]. As for smoking, the results of this study indicated that smoking and having diabetes were significantly correlated—the percentage of those who had diabetes was higher among smokers than among non-smokers. The IDF, the WHO, the ADA, and Taiwan’s Ministry of Health and Welfare have all listed smoking as one of the risk factors for developing diabetes [6,9,31]. Critical literature reviews on this topic are endless. Tonstad [44] conducted critical literature exploration research assessing the relationship among smoking, quitting smoking, and diabetes; she concluded that smoking elevates the risk for developing diabetes, but found conflicting results on whether or not quitting smoking reduces the risk of diabetes. Only after individuals had quit smoking for a certain number of years did their risk of diabetes decrease to that of non-smokers. People who quit smoking have a lower risk of diabetes than those who smoke, but former smokers’ risk levels will never be as low as those of people who have never smoked [45]. Beziaud et al. also conducted a study on a sample population of 28,409 residents aged 20–69 years old living in central and western France in 1998 concerning the effect of smoking and quitting smoking on the risk of having diabetes. Their findings showed that smoking was correlated with having diabetes, and that the risk was particularly significant for males. However, their results did not prove that smoking on a daily basis was correlated with having diabetes. Interestingly, this study also found that quitting smoking did not reduce the risk of having diabetes [45].

The reason that drinking and exercise habits appear to be insignificant may be due to both factors being self-perceived habits; there may be a gap between actual and perceived habits. In addition, the variables of frequency, intake volume, and alcohol concentration for those who drink were not determined, and therefore it is difficult to distinguish the difference between minimal or moderate consumption and alcoholism. Thus, a more comprehensive consideration of these factors in devising variable studies on this topic will produce better analytical results.

Finally, much of the research has suggested that nicotine is the major factor in smokers’ heightened risk of diabetes. In their research on the impact of nicotine on smokers, Tweed et al. [46] mentioned that nicotine decreases the sensitivity of the human body to insulin and thus increases the risk of diabetes. Also, the coexisting conditions “nicotine and diabetes” can never be negligible. Sifat et al. [47] emphasized that smoking and diabetes are generally in coexisting conditions, which will result in ischemic stroke. The coexistence of smoking and diabetes may evoke an additive effect with regard to the alterations of brain transporters. The addictive effect may deteriorate the prognosis and recovery of ischemic stroke. However, smoking is not the only source of nicotine; second-hand smoke is another source. Second-hand smoke is a mixture of two types of smoke: mainstream smoke and side-stream smoke that contains components such as NOx, nicotine, carbon monoxide, various carcinogens, and co-carcinogens. Therefore, this study specifically included passive smoke as a research variable and explored the correlation between passive smoke and having diabetes.

This research showed that everyone who had been exposed to a second-hand smoke environment had a higher rate of diabetes than those who had not been exposed, although this variable was not significant in the male population. One of the reasons for this non-significance may be that, to exclude the nicotine damage caused by being a smoker, this study excluded smokers from the study population, and the majority of smokers are male. This exclusion may have weakened the sample number of the male population and caused the non-significant result. After controlling for the demographic statistic variable, we performed a logistic regression analysis and found that second-hand smoke had a significant impact on the occurrence of diabetes, both for the overall population and for each gender.

The findings of the research corresponded with Ko et al.’s [26] and Hayashino et al.’s [27] studies, suggesting that exposure to second-hand smoke was an important risk factor for having diabetes. As for the research results regarding the long-term exposure of females to second-hand smoke, the results also corresponded with Zhang et al.’s study [28], showing that there was a significantly higher diabetes proportion among females who had been exposed to passive smoke environments. Based on the research results of the aforementioned studies, therefore, a nonsmoker’s avoidance of second-hand smoking environments can possibly help minimize the risk of diabetes.

## 5. Conclusions

The demographic variables of age, educational level, and employment had obvious impacts on the sustainable risk of developing diabetes: advancing age increases risk, while higher educational levels and employment lower it. In regard to gender, although the study showed that disease was more common among males than females, the actual factors that impact the risk of developing diabetes are behavioral rather than biological. Therefore, gender is not a factor that impacts diabetes. As for family income, the results of this study indicated that there is a correlation between family income and diabetes, but that the impact of family income is comparably low. Both the lowest and the highest income groups had higher disease risks, and many factors contributed to this result. Meanwhile, every health exam variable was significantly correlated with having diabetes. Therefore, maintaining a healthy weight and preventing hypertension can effectively reduce the sustainable risk of diabetes.

Additionally, we believe that smoking affects the risk of developing diabetes. Smokers, particularly male smokers, have a relatively higher risk of developing the disease. Finally, the results of this study revealed that second-hand smoke significantly heightened the risk of diabetes. The literature review also supported the finding that the nicotine in tobacco products is a risk factor for diabetes. Therefore, both males and females in Taiwan must pay attention to passive smoke hazards. Even though Taiwan enacted the Tobacco Hazards Prevention Act, which prohibits smoking in public places and taxes cigarettes to reduce the hazards of tobacco products, the Act does not represent a complete ban on smoking. The hazards of tobacco products and second-hand smoke still exist in private spaces.

In addition, second-hand smoking hazards are common in the region of this research, and the prevalence of smoking in the male population is also an important risk factor that affects the occurrence of diabetes. Thus, this study recommends that the relevant health authorities and hospitals actively advocate quitting smoking, and educate smokers on the considerable damage that they cause to the people around them. Furthermore, anti-smoking and second-hand smoking hazard information should be promoted, specifically among nonsmokers, so that they can avoid being exposed to second-hand smoke.

## 6. Strengths and Limitations

The strength of this study lies in that the paper went through a systematic review to investigate the diabetes variables, including demographic, health exam, healthy behaviors, and environment situations. The data was from a larger database with a pool of 6057 people. Hence, the statistical analysis has a greater accuracy and lower bias. Also, the investigation of the diabetes database maintained by a community hospital can help understand the causes of diabetes from different variables and further prompt the health promotion of the community. The findings of this study may provide some evidence for community health management and help hospitals design and develop more effective programs to prevent the occurrence of diabetes.

A limitation of the analysis is that the study did not simultaneously consider the multiple variables for the causes of diabetes. One variable may be affected by the other variables. For example, BMI may be affected by the smoking variable. Smoking may be associated with a lower body weight. There may be a kind of confounding effect in the study. To avoid the context of confounding, further research may use multivariable logistic regression to explore the causes of diabetes, using all statistically significant variables of the study. Furthermore, future study may explore the situation of mutual adjustment for each variable in the multivariable logistic model. Finally, the healthy behaviors were obtained from the samples’ self-perceived habits. Hence, while applying the results of healthy behaviors, the researchers should pay attention to some gaps between actual and perceived habits.

## Figures and Tables

**Table 1:** Correlation of Each Demographic Variable and Having Diabetes.

Variables		Not Diabetic	Diabetic	Total	Chi-square Value	*p*-Value	
Gender					4.103	0.043 *	
	Male	2348	332	2680			
	Female	2863	343	3206			
Age					215.080	<0.001 ***	
	30–44	1133	37	1170			
	45–54	1577	130	1707			
	55–64	1374	228	1602			
	65–74	553	136	689			
	75+	574	144	718			
Levels of Education					131.434	<0.001 ***	
	Illiterate	224	66	290			
	Literate or Elementary	1137	236	1373			
	Junior High	891	121	1012			
	High School	1497	127	1624			
	College Educated and Above	1157	82	1239			
Employment					125.043	<0.001 ***	
	Unemployed	2022	403	2425			
	Employed	2506	179	2685			
Family Income (NT$)					29.484	<0.001 ***	
	Under 800,000	2528	392	2920			
	800,000–1 Million	955	92	1047			
	1–1.3 Million	437	35	472			
	1.3+ Million	283	25	308			

* <0.05, *** <0.001; NT$: New Taiwan Dollar.

**Table 2:** Proportional Testing of Having Diabetes of Each Variable.

Variables		Percentage of the Population with Diabetes (%)	Test Statistics	Results
Gender			2.025683	Reject H_0_
	Male	12.39		
	Female	10.7		
Employment			11.18225	Reject H_0_
	No	16.62		
	Yes	6.67		
BMI			−9.51297	Reject H_0_
	Normal	8.24		
	Higher-than-Normal	16.31		
Waist-Hip Ratio			−14.4547	Reject H_0_
	Normal	7.86		
	Higher-than-Normal	21.46		
Body Fat Percentage			−5.29498	Reject H_0_
	Normal	9.24		
	Higher-than-Normal	13.64		
Hypertension			−8.01787	Reject H_0_
	No	10.31		
	Yes	21.08		
Smoking			−2.85275	Reject H_0_
	No	11.95		
	Yes	16.89		

H_0_: the ratios of having diabetes in both subgroups are the same.

**Table 3:** Correlation of Each Health Exam Variable and Having Diabetes.

Variables		Not Diabetic	Diabetic	Total	Chi-square Value	*p*-Value	
BMI					90.497	<0.001 ***	
	Normal	3240	291	3531			
	Higher-than-Normal	1971	384	2355			
Waist-Hip Ratio					208.939	<0.001 ***	
	Normal	3985	340	4325			
	Higher-than-Normal	1226	335	1561			
Body Fat Percentage					28.037	<0.001 ***	
	Normal	2641	269	2910			
	Higher-than-Normal	2570	406	2976			
Hypertension					64.286	<0.001 ***	
	No	4713	542	5255			
	Yes	498	133	631			

*** <0.001; BMI normal range: <24 kg/m^2^; Waist-Hip Ratio normal range: male < 0.9, female < 0.8; Body Fat Percentage normal range: male 17–23%, female 20–27%.

**Table 4:** Correlation of Each Healthy Behaviors Variable and Having Diabetes.

Variables		Not Diabetic	Diabetic	Total	Chi-Square Value	*p*-Value	
Exercise Habits					0.000	0.999	
	No	1964	259	2223			
	Yes	2919	385	3304			
Smoking Habits					8.138	0.004 **	
	No	1894	257	2151			
	Yes	374	76	450			
Drinking Habits					0.091	0.762	
	No	4637	600	5237			
	Yes	508	63	571			

** <0.01.

**Table 5:** Proportion Test of Having Diabetes for Each Variable.

Variables		Proportion of Having Diabetes	Test Statistics	Results
Second-hand Smoking Hazards (Overall Sample)			−2.128	Reject H_0_
	No	11.95		
	Yes	14.17		
Second-hand Smoking Hazards (Male Sample)			−0.776	Accept H_0_
	No	13.89		
	Yes	15.36		
Second-hand Smoking Hazards (Female Sample)			−2.254	Reject H_0_
	No	10.83		
	Yes	13.65		

H_0_: the proportion having diabetes in both subgroups is the same.

**Table 6:** Regression Analysis for Each Variable and Having Diabetes.

Variables		Odds Ratio	95% Confidence Intervals	*p*-Value
Passive Smoke Hazards (Overall Sample)				
	No (Reference Group)			
	Yes	1.389	(1.172–1.647)	<0.001 ***
Passive Smoke Hazards (Male Sample)				
	No (Reference Group)			
	Yes	1.301	(1.034–1.636)	0.024 *
Passive Smoke Hazards (Female Sample)				
	No (Reference Group)			
	Yes	1.532	(1.144–2.053)	0.004 **

* <0.05, ** <0.01, *** <0.001.
